# Cyclic voltammetry data of polypyridine ligands and Co(II)-polypyridine complexes

**DOI:** 10.1016/j.dib.2018.12.043

**Published:** 2018-12-16

**Authors:** Hendrik Ferreira, Marrigje M. Conradie, Jeanet Conradie

**Affiliations:** Department of Chemistry, University of the Free State, PO Box 339, Bloemfontein, 9300, South Africa

## Abstract

The data presented in this article is related to the research article entitled “Electrochemical and electronic properties of a series of substituted polypyridine ligands and their Co(II) complexes” (Ferreira et al., 2019). This data article presents electrochemical data of five polypyridine ligands, as well as of the three redox couples of each of their corresponding five polypyridine-containing Co(II) complexes. All complexes exhibit two Co-based redox couples (Co^III/II^ and Co^II/I^), as well as a ligand-based reduction of the Co(I) complex.

**Specifications table**

**Table:** 

Subject area	*Chemistry*
More specific subject area	*Electrochemistry*
Type of data	*Table, text file, graph, figure*
How data were acquired	*BAS 100B/W electrochemical analyzer (Electrochemical studies).*
Data format	*Raw and analyzed.*
Experimental factors	*Samples were used as synthesized. The solvent-electrolyte solution in the electrochemical cell was degassed with Ar for 10 min, the sample was added, the sample-solvent-electrolyte solution was then degassed for another 2 min and the cell was kept under a blanket of purified argon during the electrochemical experiments.*
Experimental features	*All electrochemical experiments were done in a 2 ml electrochemical cell containing three-electrodes (a glassy carbon working electrode, a Pt auxiliary electrode and a Ag/Ag*^*+*^*reference electrode), connected to a BAS 100B/W electrochemical analyzer. Data obtained was exported to excel for analysis and diagram preparation.*
Data source location	*Department of Chemistry, University of the Free State, Nelson Mandela Street, Bloemfontein, South Africa.*
Data accessibility	*Data is with article.*
Related research article	*Hendrik Ferreira, Marrigje M. Conradie and Jeanet Conradie, Electrochemical and electronic properties of a series of substituted polypyridine ligands and their Co(II) complexes, Inorganica Chimica Acta,* 2019, 486, 26–35. DOI 10.1016/j.ica.2018.10.020 [1].

**Value of the data**

• This data provides cyclic voltammograms for five polypyridine ligands, 2,2′:6′,2″-terpyridine (tpy, ligand **1a**), 2,2′-dipyridyl (bpy, ligand **2a**), 4,4′-dimethyl-2,2′-bipyridine (4,4′-di-Me-bpy, ligand **3a**), 4,4′-di-tert-butyl-2,2′-dipyridyl (4,4′-di-^*t*^Bu-bpy, ligand **4a**) and 4,4′-dimethoxy-2,2′-bipyridine (4,4′-di-OMe-bpy, ligand **5a**).
• This data provides cyclic voltammograms and detailed electrochemical data for Co(tpy)_2_(NO_3_)_2_, complex **1**, Co(bpy)_3_(NO_3_)_2_, complex **2**, Co(4,4′-di-Me-bpy)_3_(NO_3_)_2_, complex **3**, Co(4,4′-di-*^t^*Bu-bpy)_3_(NO_3_)_2_, complex **4** and Co(4,4′-di-OMe-bpy)(NO_3_)_2_, complex **5**.
• The current contribution is the first to present complete electrochemical data for all three reversible redox peaks at different scan rates, over two orders of magnitudes, for terpyridine-Co(II), bipyridine-Co(II), as well as substituted bipyridine-Co(II) complexes.
• Accurate redox data is important to determine the potential of a compound, in order to determine its suitability to act as a redox mediator, to be used in dye-sensitized solar cells (DSSC) [2], [3], [4].

## 1 Data

**Fig. 1:**
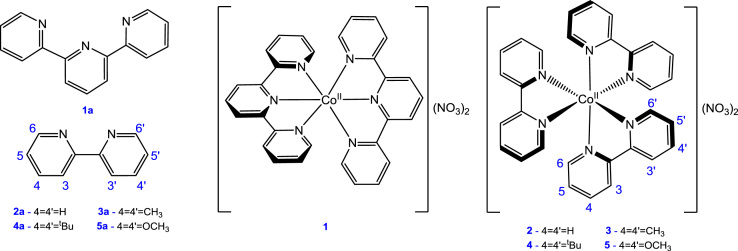
Structure and numbering of the terpyridine (**1a**) and substituted bipyridine (**2a–5a**) ligands, as well as the terpyridine-Co(II) complex **1** and substituted bipyridine-Co(II) complexes, **2–5**.

**Fig. 2:**
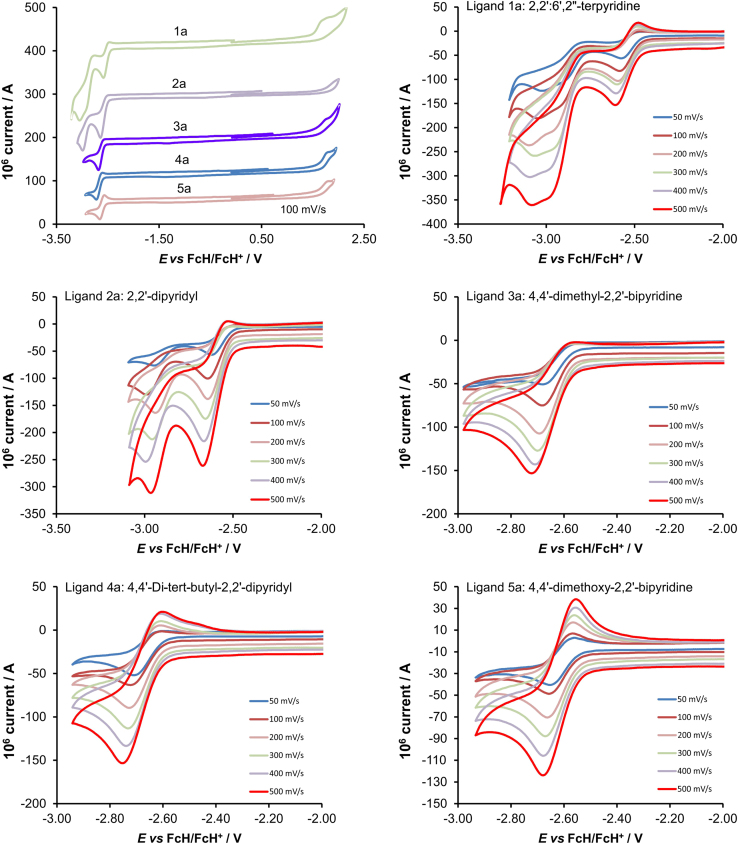
Cyclic voltammograms of *ca.* 0.002 mol dm^−3^ or saturated solutions of the free ligands **1a–5a**, at the indicated scan rates (0.10 V s^−1^ top left for the comparative graph and 0.05–0.50 V s^−1^ for all other graphs). The reduction peak of ligand **5a** is chemically (i_pa_/i_pc_ = 0.9) and electrochemically (ΔEp = E_pa_–E_pc_ = 0.088 V) reversible at all scan rates above 0.05 V s^−1^, while the reduction of free ligands **2a–5a1a – 4a** is irreversible at low scan rates.

**Fig. 3:**
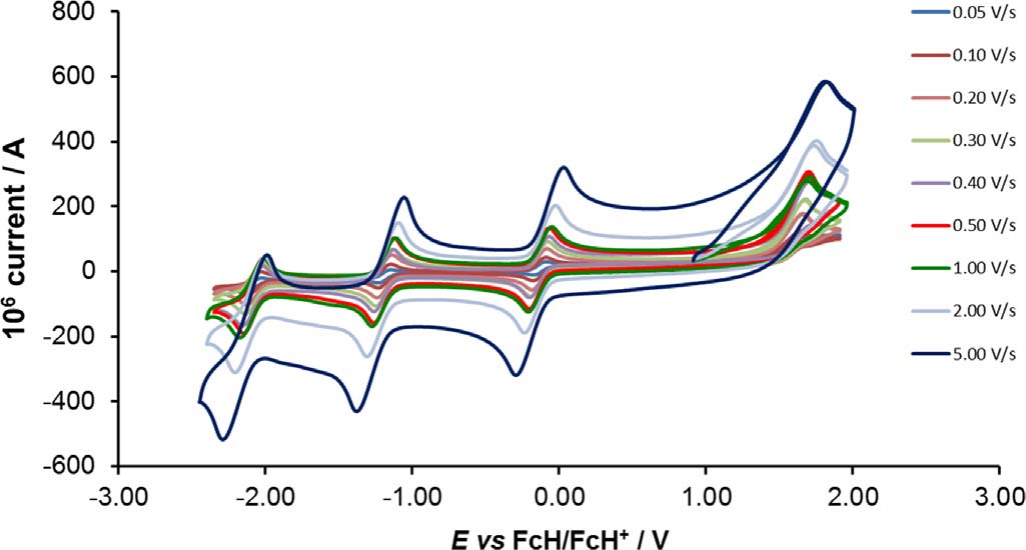
Cyclic voltammograms of complex 1, [Co(tpy)_2_](NO_3_)_2_, at scan rates of 0.05 V s^−1^ (lowest peak current) −5.00 V s^−1^ to 5.00 V s^−1^ (highest peak current). All scans were initiated in the positive direction from 1 V. Data is summarized in Table 1.

**Fig. 4:**
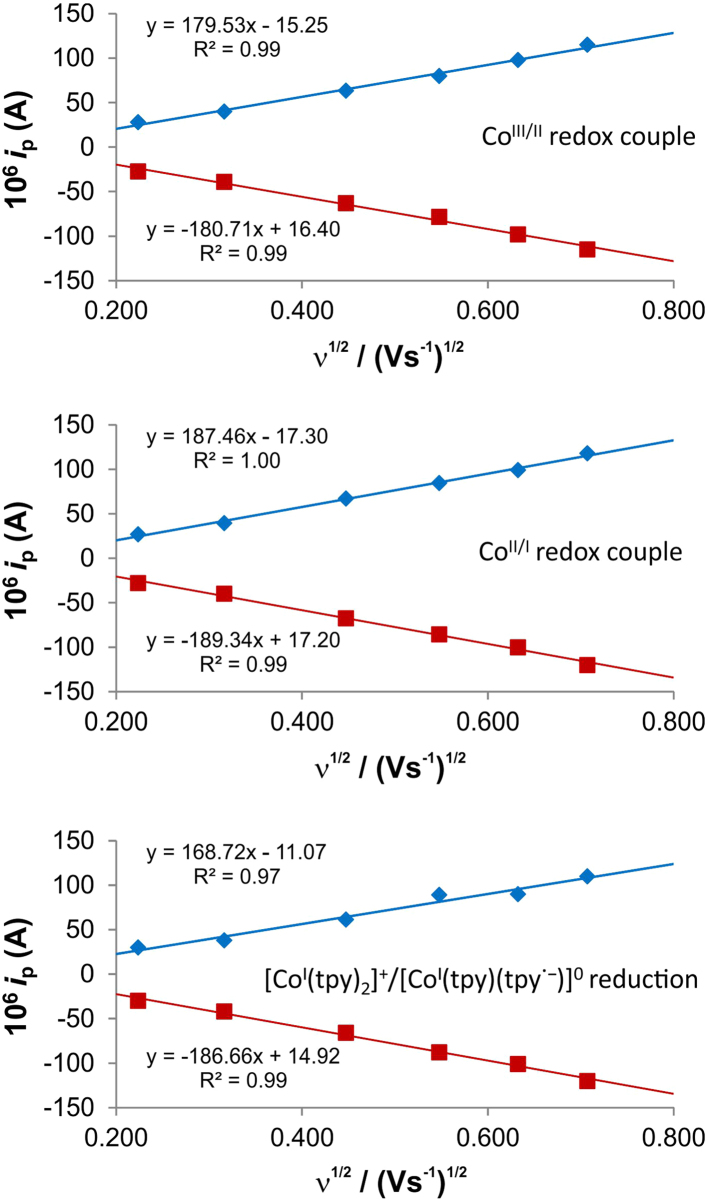
The linear relationship between the peak currents (*i*_p_) *vs* the square root of the scan rate (ν^½^) for the three main redox events, in the CV of [Co(tpy)_2_](NO_3_)_2_ (complex **1**) in Fig. 3. This relationship can be described by the linear Randles–Sevcik equation $i_{p}=(2.69{\mathrm{X10}}^{5})n^{1.5}{\mathrm{AD}}^{0.5}C\nu^{0.5}$ (*n* = the number of exchanged electrons, *A* = electrode area (cm^2^), *D* = diffusion coefficient (cm^2^ s^−1^), *C* = bulk concentration (mol cm^−3^) of the electroactive species.

**Fig. 5:**
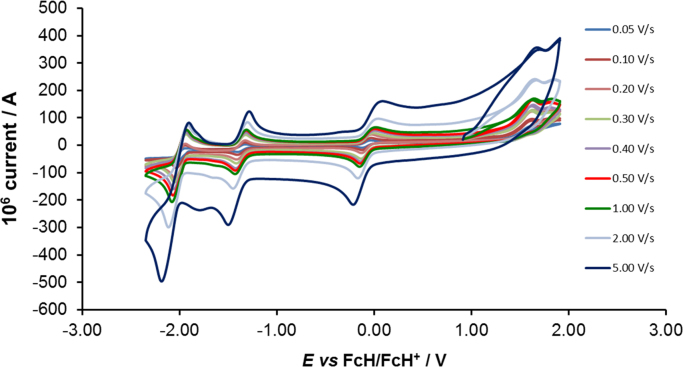
Cyclic voltammograms of [Co(bpy)_2_](NO_3_)_2_ (complex **2**), at scan rates of 0.05 V s^−1^ (lowest peak current) −5.00 V s^−1^ to 5.00 V s^−1^ (highest peak current). All scans were initiated in the positive direction from 1 V. Data is summarized in Table 2.

**Fig. 6:**
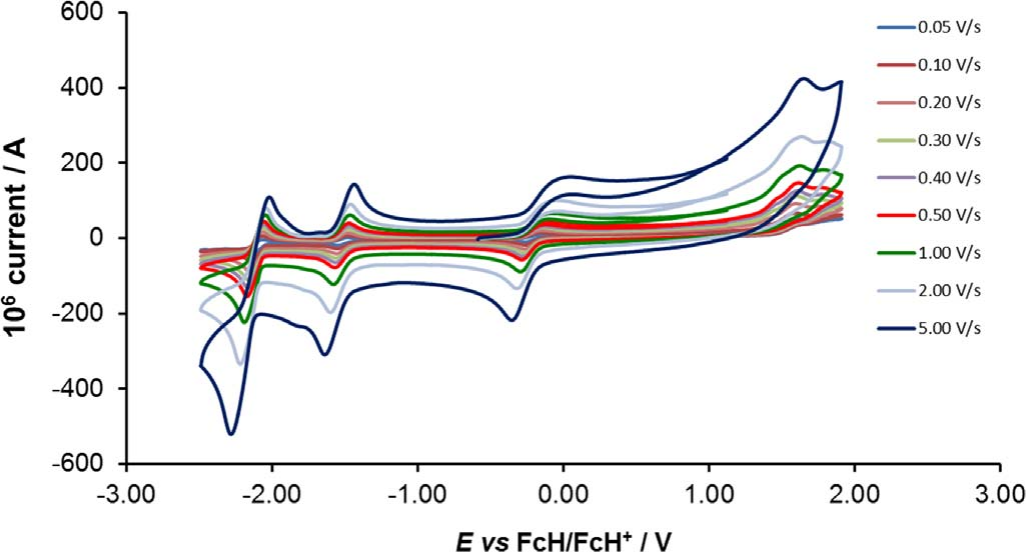
Cyclic voltammograms of [Co(4,4′-Me-bpy)_2_](NO_3_)_2_ (complex **3**), at scan rates of 0.05 V s^−1^ (lowest peak current) −5.00 V s^−1^ to 5.00 V s^−1^ (highest peak current). All scans were initiated in the positive direction from 1 V. Data is summarized in Table 3.

**Fig. 7:**
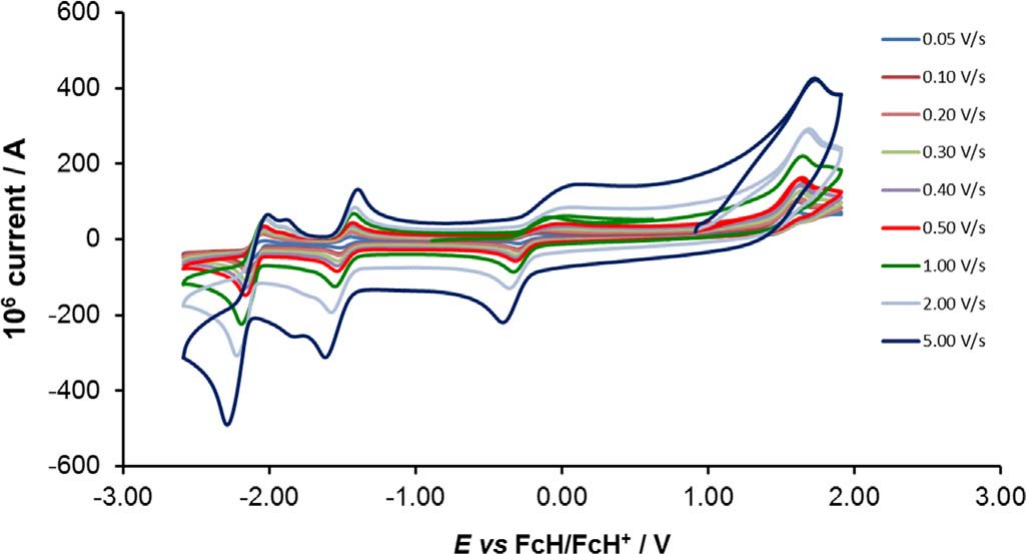
Cyclic voltammograms of [Co(4,4′-^t^Bu-bpy)_2_](NO_3_)_2_ (complex **4**), at scan rates of 0.05 V s^−1^ (lowest peak current)−5.00 V s^−1^ to 5.00 V s^−1^ (highest peak current). All scans were initiated in the positive direction from 1 V. Data is summarized in Table 4.

**Fig. 8:**
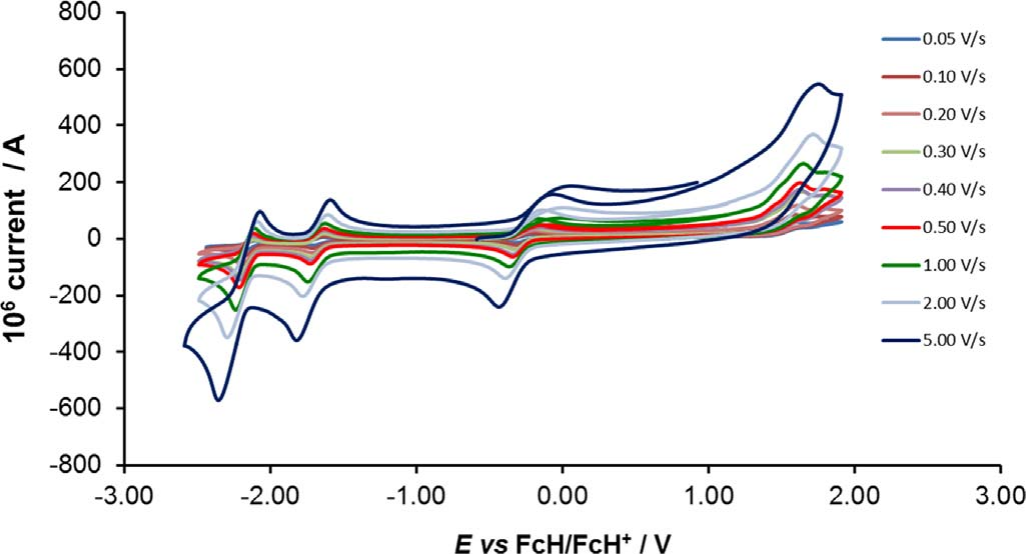
Cyclic voltammograms of [Co(4,4′-OMe-bpy)_2_](NO_3_)_2_ (complex **5**), at scan rates of 0.05 V s^−1^ (lowest peak current) −5.00 V s^−1^ to 5.00 V s^−1^ (highest peak current). All scans were initiated in the positive direction from 1 V. Data is summarized in Table 5.

Fig. 1 gives the structures of ligands **1a**–**5a** and complexes **1**–**5**. Fig. 2 shows the cyclic voltammetry (CV) scans for the polypyridyl free ligands **1a**–**5a** at different scan rates (0.10 V s^−^^1^ scans from [1]). Cyclic voltammograms of the complexes **1**–**5**, showing four redox events each, are presented in Fig. 3, Fig. 4, Fig. 5, Fig. 6, Fig. 7, Fig. 8 (0.10 V s^−1^ scans from [1]), with the data summarized in Table 1, Table 2, Table 3, Table 4, Table 5. The redox events are the Co^III/II^ redox couple (peak 1), the Co^II/I^ redox couple (peak 2) and the ligand reduction peak (peak 3), as well as an irreversible peak at *ca*. 1.63 V *vs* FcH/FcH^+^ (preliminary assigned to anionic nitrate oxidation). The data obtained in this data article, compares well with available published data on some of the redox events for some of the complexes, namely complex **1** [5], [6], [7], [8], [9], [10], complex **2** [11], [12], [13] and complex **5** [11]; obtained under different experimental conditions (different solvents, scan rates and supporting electrolytes). The linear responses obtained for the graphs of the peak currents *vs* the square root of the scan rate, for three main redox events in the CV of complex **1** (see Fig. 4), are in agreement with the Randles–Sevcik equation [14].

**Table 1:** Electrochemical data (potential in V *vs* FcH/FcH^+^ and current in A) obtained in CH_3_CN for *ca*. 0.002 mol dm^−3^ of [Co(tpy)_2_](NO_3_)_2_ (complex **1**), at indicated scan rates in V s^−1^. Peak 1 is the Co^III/II^ redox couple, peak 2 the Co^II/I^ redox couple and peak 3 the ligand reduction peak.Pl

	Scan rate	*E*_pa_	*E*_pc_	*E*°′	ΔE	10^6^*I*_pa_	*I*_pc_/*I*_pa_
Peak 1	0.05	−0.090	−0.174	−0.132	0.084	28.0	1.0
	0.10	−0.094	−0.178	−0.136	0.084	40.0	1.0
	0.20	−0.088	−0.184	−0.136	0.096	63.5	1.0
	0.30	−0.080	−0.192	−0.136	0.112	80.0	1.0
	0.40	−0.072	−0.208	−0.140	0.136	98.0	1.0
	0.50	−0.072	−0.202	−0.137	0.130	115.0	1.0
	1.00	−0.054	−0.208	−0.131	0.154	113.0	1.0
	2.00	−0.030	−0.234	−0.132	0.204	165.0	1.0
	5.00	0.026	−0.294	−0.134	0.320	248.0	1.0
Peak 2	0.05	−1.148	−1.230	−1.189	0.082	27.0	1.0
	0.10	−1.146	−1.232	−1.189	0.086	39.5	1.0
	0.20	−1.140	−1.240	−1.190	0.100	67.0	1.0
	0.30	−1.134	−1.248	−1.191	0.114	84.5	1.0
	0.40	−1.130	−1.262	−1.196	0.132	99.0	1.0
	0.50	−1.126	−1.262	−1.194	0.136	118.0	1.0
	1.00	−1.116	−1.274	−1.195	0.158	115.0	1.0
	2.00	−1.098	−1.308	−1.203	0.210	168.0	1.0
	5.00	−1.058	−1.378	−1.218	0.320	263.0	1.0
Peak 3	0.05	−2.028	−2.120	−2.074	0.092	30.0	1.0
	0.10	−2.030	−2.120	−2.075	0.090	38.0	1.1
	0.20	−2.022	−2.128	−2.075	0.106	61.5	1.1
	0.30	−2.020	−2.138	−2.079	0.118	89.0	1.0
	0.40	−2.020	−2.154	−2.087	0.134	90.0	1.1
	0.50	−2.014	−2.148	−2.081	0.134	110.0	1.1
	1.00	−2.012	−2.174	−2.093	0.162	110.0	1.1
	2.00	−2.006	−2.208	−2.107	0.202	155.0	1.1
	5.00	−1.990	−2.290	−2.140	0.300	205.0	1.2

**Table 2:** Electrochemical data (potential in V *vs* FcH/FcH^+^ and current in A) obtained in CH_3_CN for *ca*. 0.002 mol dm^−3^ of [Co(bpy)_2_](NO_3_)_2_ (complex **2**), at indicated scan rates in V s^−1^. Peak 1 is the Co^III/II^ redox couple, peak 2 the Co^II/I^ redox couple and peak 3 the ligand reduction peak.

	Scan rate	*E*_pa_	*E*_pc_	*E*°׳	ΔE	10^6^*I*_pa_	*I*_pc_/*I*_pa_
Peak 1	0.05	−0.038	−0.120	−0.079	0.082	16.5	1.0
	0.10	−0.038	−0.124	−0.081	0.086	23.0	1.1
	0.20	−0.032	−0.128	−0.080	0.096	34.0	1.1
	0.30	−0.016	−0.134	−0.075	0.118	36.5	1.2
	0.40	−0.002	−0.140	−0.071	0.138	43.0	1.2
	0.50	0.000	−0.144	−0.072	0.144	46.0	1.2
	1.00	0.008	−0.150	−0.071	0.158	49.0	1.2
	2.00	0.034	−0.168	−0.067	0.202	66.0	1.4
	5.00	0.086	−0.216	−0.065	0.302	100.0	1.4
Peak 2	0.05	−1.332	−1.402	−1.367	0.070	16.5	1.1
	0.10	−1.334	−1.404	−1.369	0.070	23.5	0.9
	0.20	−1.330	−1.412	−1.371	0.082	35.0	1.1
	0.30	−1.324	−1.416	−1.370	0.092	40.0	1.2
	0.40	−1.320	−1.420	−1.370	0.100	45.0	1.3
	0.50	−1.318	−1.426	−1.372	0.108	55.0	1.2
	1.00	−1.316	−1.428	−1.372	0.112	56.0	1.2
	2.00	−1.304	−1.448	−1.376	0.144	83.0	1.2
	5.00	−1.288	−1.500	−1.394	0.212	120.0	1.3
Peak 3	0.05	−1.950	−2.028	−1.989	0.078	33.5	1.1
	0.10	−1.946	−2.032	−1.989	0.086	46.5	1.2
	0.20	−1.944	−2.048	−1.996	0.104	57.0	1.5
	0.30	−1.934	−2.052	−1.993	0.118	83.0	1.2
	0.40	−1.932	−2.062	−1.997	0.130	96.0	1.2
	0.50	−1.930	−2.066	−1.998	0.136	110.0	1.2
	1.00	−1.928	−2.078	−2.003	0.150	118.0	1.2
	2.00	−1.920	−2.116	−2.018	0.196	156.0	1.3
	5.00	−1.906	−2.186	−2.046	0.280	230.0	1.3

**Table 3:** Electrochemical data (potential in V *vs* FcH/FcH^+^ and current in A) obtained in CH_3_CN for *ca*. 0.002 mol dm^−3^ of [Co(4,4׳-Me-bpy)_2_](NO_3_)_2_ (complex **3**), at indicated scan rates in V s^−1^. Peak 1 is the Co^III/II^ redox couple, peak 2 the Co^II/I^ redox couple and peak 3 the ligand reduction peak.

	Scan rate	*E*_pa_	*E*_pc_	*E*°׳	ΔE	10^6^*I*_pa_	*I*_pc_/*I*_pa_
Peak 1	0.05	−0.166	−0.262	−0.214	0.096	10.0	1.2
	0.10	−0.162	−0.264	−0.213	0.102	15.0	1.2
	0.20	−0.152	−0.268	−0.210	0.116	20.0	1.4
	0.30	−0.146	−0.274	−0.210	0.128	25.5	1.4
	0.40	−0.132	−0.276	−0.204	0.144	28.5	1.5
	0.50	−0.132	−0.284	−0.208	0.152	33.0	1.4
	1.00	−0.104	−0.296	−0.200	0.192	44.0	1.6
	2.00	−0.058	−0.318	−0.188	0.260	58.0	1.7
	5.00	0.040	−0.352	−0.156	0.392	95.0	1.5
Peak 2	0.05	−1.482	−1.546	−1.514	0.064	11.5	1.0
	0.10	−1.486	−1.548	−1.517	0.062	16.0	1.1
	0.20	−1.482	−1.554	−1.518	0.072	22.5	1.2
	0.30	−1.480	−1.562	−1.521	0.082	28.0	1.2
	0.40	−1.476	−1.564	−1.520	0.088	34.0	1.2
	0.50	−1.478	−1.568	−1.523	0.090	40.0	1.2
	1.00	−1.468	−1.578	−1.523	0.110	57.0	1.3
	2.00	−1.464	−1.598	−1.531	0.134	88.0	1.4
	5.00	−1.440	−1.636	−1.538	0.196	155.0	1.0
Peak 3	0.05	−2.068	−2.138	−2.103	0.070	23.0	1.2
	0.10	−2.070	−2.144	−2.107	0.074	340.0	1.2
	0.20	−2.066	−2.154	−2.110	0.088	50.5	1.2
	0.30	−2.062	−2.164	−2.113	0.102	65.0	1.2
	0.40	−2.056	−2.166	−2.111	0.110	75.0	1.2
	0.50	−2.058	−2.172	−2.115	0.114	90.0	1.2
	1.00	−2.048	−2.190	−2.119	0.142	123.0	1.2
	2.00	−2.042	−2.220	−2.131	0.178	174.0	1.2
	5.00	−2.022	−2.280	−2.151	0.258	240.0	1.4

**Table 4:** Electrochemical data (potential in V *vs* FcH/FcH^+^ and current in A) obtained in CH_3_CN for *ca*. 0.002 mol dm^−3^ of [Co(4,4׳-^t^Bu-bpy)_2_](NO_3_)_2_ (complex **4**), at indicated scan rates in V s^−1^. Peak 1 is the Co^III/II^ redox couple, peak 2 the Co^II/I^ redox couple and peak 3 the ligand reduction peak.

	Scan rate	*E*_pa_	*E*_pc_	*E*°׳	ΔE	10^6^*I*_pa_	*I*_pc_/*I*_pa_
Peak 1	0.05	−0.184	−0.294	−0.239	0.110	13.0	1.2
	0.10	−0.174	−0.302	−0.238	0.128	20.5	1.2
	0.20	−0.144	−0.298	−0.221	0.154	21.0	1.4
	0.30	−0.044	−0.310	−0.177	0.266	21.5	1.6
	0.40	0.020	−0.312	−0.146	0.332	25.0	1.6
	0.50	0.002	−0.316	−0.157	0.318	28.0	1.6
	1.00	−0.088	−0.328	−0.208	0.240	55.0	1.3
	2.00	0.050	−0.358	−0.154	0.408	48.0	1.8
	5.00	0.114	−0.402	−0.144	0.516	53.0	2.4
Peak 2	0.05	−1.448	−1.516	−1.482	0.068	14.0	0.9
	0.10	−1.448	−1.520	−1.484	0.072	24.0	1.1
	0.20	−1.444	−1.520	−1.482	0.076	25.0	1.2
	0.30	−1.440	−1.530	−1.485	0.090	32.0	1.2
	0.40	−1.434	−1.532	−1.483	0.098	36.0	1.3
	0.50	−1.434	−1.536	−1.485	0.102	43.0	1.3
	1.00	−1.426	−1.548	−1.487	0.122	65.0	1.3
	2.00	−1.418	−1.574	−1.496	0.156	82.0	1.4
	5.00	−1.400	−1.616	−1.508	0.216	126.0	1.3
Peak 3	0.05	−2.034	−2.130	−2.082	0.096	28.0	1.3
	0.10	−2.046	−2.140	−2.093	0.094	42.5	1.3
	0.20	−2.046	−2.146	−2.096	0.100	45.0	1.4
	0.30	−2.040	−2.156	−2.098	0.116	56.0	1.4
	0.40	−2.038	−2.162	−2.100	0.124	67.0	1.3
	0.50	−2.038	−2.164	−2.101	0.126	75.0	1.4
	1.00	−2.028	−2.190	−2.109	0.162	115.0	1.3
	2.00	−2.028	−2.222	−2.125	0.194	136.0	1.4
	5.00	−2.012	−2.288	−2.150	0.276	195.0	1.5

**Table 5:** Electrochemical data (potential in V *vs* FcH/FcH^+^ and current in A) obtained in CH_3_CN for *ca*. 0.002 mol dm^−3^ of [Co(4,4׳-OMe-bpy)_2_](NO_3_)_2_ (complex **5**), at indicated scan rates in V s^−1^. Peak 1 is the Co^III/II^ redox couple, peak 2 the Co^II/I^ redox couple and peak 3 the ligand reduction peak.

	Scan rate	*E*_pa_	*E*_pc_	*E*°׳	ΔE	10^6^*I*_pa_	*I*_pc_/*I*_pa_
Peak 1	0.05	−0.218	−0.314	−0.266	0.096	13.0	1.2
	0.10	−0.216	−0.316	−0.266	0.100	19.0	1.2
	0.20	−0.210	−0.324	−0.267	0.114	29.5	1.2
	0.30	−0.192	−0.330	−0.261	0.138	33.0	1.3
	0.40	−0.196	−0.338	−0.267	0.142	40.0	1.3
	0.50	−0.192	−0.340	−0.266	0.148	47.0	1.2
	1.00	−0.170	−0.366	−0.268	0.196	64.0	1.3
	2.00	−0.146	−0.390	−0.268	0.244	93.0	1.2
	5.00	−0.072	−0.434	−0.253	0.362	135.0	1.2
Peak 2	0.05	−1.640	−1.706	−1.673	0.066	13.0	1.1
	0.10	−1.638	−1.708	−1.673	0.070	18.0	1.2
	0.20	−1.638	−1.714	−1.676	0.076	22.5	1.5
	0.30	−1.634	−1.728	−1.681	0.094	37.5	1.1
	0.40	−1.636	−1.724	−1.680	0.088	34.0	1.6
	0.50	−1.632	−1.728	−1.680	0.096	40.0	1.5
	1.00	−1.628	−1.744	−1.686	0.116	53.0	2.0
	2.00	−1.608	−1.778	−1.693	0.170	87.0	1.5
	5.00	−1.588	−1.818	−1.703	0.230	120.0	1.8
Peak 3	0.05	−2.084	−2.178	−2.131	0.094	14.0	1.7
	0.10	−2.102	−2.180	−2.141	0.078	17.0	2.3
	0.20	−2.122	−2.196	−2.159	0.074	27.5	2.3
	0.30	−2.118	−2.216	−2.167	0.098	48.0	1.7
	0.40	−2.120	−2.210	−2.165	0.090	51.0	2.0
	0.50	−2.116	−2.214	−2.165	0.098	65.0	1.8
	1.00	−2.110	−2.238	−2.174	0.128	99.0	1.6
	2.00	−2.090	−2.296	−2.193	0.206	156.0	1.4
	5.00	−2.072	−2.356	−2.214	0.284	250.0	1.4

## 2 Experimental design, materials, and methods

Electrochemical studies, by means of cyclic voltammetry (CV), were performed at 25 °C on a BAS 100B/W electrochemical analyser under inert conditions as described previously [1]. The concentration of the analyte was 0.002 mol dm^−3^ or saturated. The solvent was dry acetonitrile and the supporting electrolyte 0.1 mol dm^−3^ tetra-*n*-butylammoniumhexafluorophosphate ([^n^(Bu_4_)N][PF_6_]). A three-electrode cell comprising of a glassy carbon (surface area 7.07 × 10^−6^ m^2^) working electrode, Pt auxiliary electrode and a Ag/Ag^+^ (0.010 mol dm^−3^ AgNO_3_ in CH_3_CN) reference electrode [15], mounted on a Luggin capillary [16] was used.
